# A new species of *Hemibrycon* (Characiformes, Characidae) from the upper San Juan River drainage, Pacific versant, Colombia

**DOI:** 10.3897/zookeys.454.6954

**Published:** 2014-11-14

**Authors:** César Román-Valencia, Raquel I. Ruiz-C, Donald C. Taphorn, Carlos A. García-Alzate

**Affiliations:** 1 Universidad del Quindío, Laboratorio de Ictiología, A. A. 2639, Armenia, Colombia; 2 1822 N. Charles St., Belleville, IL, 62221 USA; 3 Universidad del Atlántico, Programa de Biología, Barranquilla, Colombia

**Keywords:** Biodiversity, taxonomy, tropical fish, new taxon

## Abstract

*Hemibrycon sanjuanensis*, new species, is described from the upper San Juan River drainage, Pacific versant, Colombia. It is distinguished from *Hemibrycon boquiae*, *Hemibrycon brevispini*, *Hemibrycon cairoense*, *Hemibrycon colombianus*, *Hemibrycon mikrostiktos*, *Hemibrycon metae*, *Hemibrycon palomae*, *Hemibrycon rafaelense* and *Hemibrycon tridens* by the presence of a circular or oblong humeral spot that is located two scales posterior to the opercle (vs. 3–4 scales in *Hemibrycon palomae*, *Hemibrycon rafaelense*, *Hemibrycon brevispini* and *Hemibrycon cairoense*, and 0–1 scales, in *Hemibrycon metae* and *Hemibrycon boquiae*). It further differs from *Hemibrycon colombianus* in having a round or oblong humeral spot (vs. rectangular). It differs from *Hemibrycon beni*, *Hemibrycon dariensis*, *Hemibrycon divisorensis*, *Hemibrycon helleri*, *Hemibrycon huambonicus*, *Hemibrycon inambari*, *Hemibrycon jabonero*, *Hemibrycon jelskii*, *Hemibrycon mikrostiktos*, *Hemibrycon polyodon*, *Hemibrycon quindos*, *Hemibrycon raqueliae*, *Hemibrycon santamartae*, *Hemibrycon surinamensis*, *Hemibrycon taeniurus*, *Hemibrycon tridens*, and *Hemibrycon yacopiae* in having melanophores on the posterior margins of the scales along the sides of body (vs. lacking melanophores on margins of scales along entire length of the sides of body). The new species differs from all congeners mentioned above in having, among other features, six teeth in the outer premaxillary row arranged in a straight line (vs. five or fewer teeth not arranged in straight line except *Hemibrycon cairoense* with two to six teeth in the outer premaxillary row).

## Abstract (es)

*Hemibrycon sanjuanensis*, ﻿nueva especie, se describe de la cuenca alta del Rio San Juan, vertiente Pacifico, Colombia. Este se distingue de *Hemibrycon boquiae*, *Hemibrycon brevispini*, *Hemibrycon cairoense*, *Hemibrycon colombianus*, *Hemibrycon mikrostiktos*, *Hemibrycon metae*, *Hemibrycon palomae*, *Hemibrycon rafaelense* y *Hemibrycon tridens* por la presencia de una mancha humeral circular u ovalada que se localiza a dos escamas posterior al borde del opérculo (vs. 3–4 escamas en *Hemibrycon palomae*, *Hemibrycon rafaelense*, *Hemibrycon brevispini* y *Hemibrycon cairoense*, y cero o una escama desde el opérculo en *Hemibrycon metae* y *Hemibrycon boquiae*). Esta se distingue de *Hemibrycon colombianus* en poseer una mancha humeral redondeada u ovalada (vs. rectangular). Esta se diferencia de *Hemibrycon beni*, *Hemibrycon dariensis*, *Hemibrycon divisorensis*, *Hemibrycon helleri*, *Hemibrycon huambonicus*, *Hemibrycon inambari*, *Hemibrycon jabonero*, *Hemibrycon jelskii*, *Hemibrycon mikrostiktos*, *Hemibrycon polyodon*, *Hemibrycon quindos*, *Hemibrycon raqueliae*, *Hemibrycon santamartae*, *Hemibrycon surinamensis*, *Hemibrycon taeniurus*, *Hemibrycon tridens*, y *Hemibrycon yacopiae* en presentar melanóforos sobre el borde posterior de las escamas a lo largo de los lados del cuerpo (vs. ausencia de melanóforos sobre el borde posterior de las escamas de las escamas en los lados del cuerpo). La nueva especie se distingue de todos los congéneres, citados arriba, por presentar seis dientes en la fila externa del premaxilar ordenados en línea recta (vs. cinco o menos no ordenados en línea recta, excepto *Hemibrycon cairoense* con dos a seis dientes en la fila externa del premaxilar).

## Introduction

Today there are 36 species reported in the genus *Hemibrycon* (Eschmeyer and Fricke 2013). Of these, 21 species are distributed in Colombian watersheds, but only one species, *Hemibrycon dariensis*, ﻿has been reported from the San Juan drainage on the Pacific coast. The present day distribution of *Hemibrycon* in Colombia suggests that this genus has its greatest diversity in Andean streams and high mountain habitats. But there are exceptions such as *Hemibrycon dariensis*, ﻿which occurs in Pacific drainages, *Hemibrycon santamartae* and *Hemibrycon* sp. n. found in northern Colombia, and *Hemibrycon metae* from the upper Meta River drainage in the Orinoco River Basin (Bertaco and Malabarba 2010; Román-Valencia and Ruiz-C 2007; Román-Valencia et al. 2009a; Román-Valencia et al. 2013). The discovery of a new species of *Hemibrycon* from the upper San Juan River Basin, Pacific versant in Colombia, is a result of the authors’ ongoing revision of *Hemibrycon* (Román-Valencia et al. 2010; 2013) and is further proof of undocumented biodiversity in the genus.

## Material and methods

Fishes were captured using seines and were preserved in the field with 10% formalin and later stored in 70% ethanol. Measurements and counts follow Armbruster (2012) and Román-Valencia et al. (2010). Measurements were made with digital calipers to 0.01 mm precision and are expressed as percentages of standard length (SL) and head length (HL). In reporting counts, values for the holotype are indicated with an asterisk (*). In the list of paratypes, the number of individuals is given immediately after the catalog number, which is followed by the range of SL in mm for that lot. Counts and measurements were taken on the left side of specimens when possible. A multivariate analysis was performed on the morphometric data with the PAST program, version 1.81 for Windows (Hammer et al. 2008), and a variable canonical analysis (CVA) was undertaken assuming allometric growth. All measurements were log transformed, correcting for size using Burnaby methods (Burnaby 1966) in the software PAST to adjust for or eliminate the influence of size and compensate for the allometric growth. In the CVA we included data for *Hemibrycon dariensis* (n=71), which is allopatric with the new species in the San Juan River drainage and *Hemibrycon cairoense* (n=46), which occurs in nearby watersheds. Morphological analysis based on bivariate or multivariate testing seeks to establish parameters (= morphological characters) for discriminating the closest species to the new taxon from other species present in the area of geographic distribution.

Osteological observations were made on cleared and stained specimens (C and S) prepared according to Taylor and Van Dyke (1985) and Song and Parenti (1995). Bone nomenclature follows Weitzman (1962), Vari (1995), and Ruiz-C and Román-Valencia (2006). Specimens are deposited in the Ichthyology Laboratory at the Universidad del Quindío, Armenia, Colombia (IUQ). Institutional acronyms for comparative material follow Sabaj-Pérez (2010). All collections were made in Colombia.

## Taxonomy

### Hemibrycon sanjuanensis sp. n.

Taxon classificationAnimaliaCharaciformesCharacidae

http://zoobank.org/5BF13995-FCD7-42FD-93E3-9B3F62A1BF80

**Figure 1.**
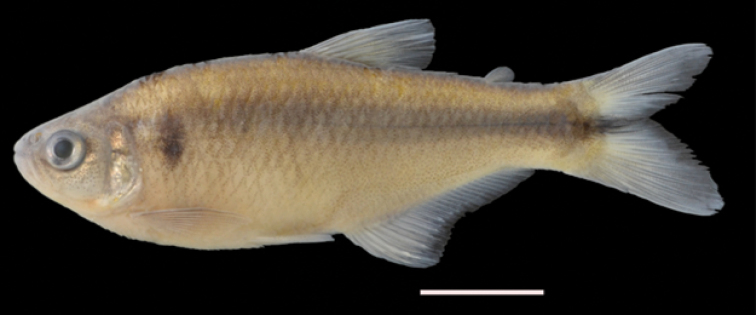
*Hemibrycon sanjuanensis* sp. n., holotype IUQ 3693. 53.5 mm SL, Colombia, Risaralda State, Pueblo Rico Municipality, El Recreo, upper San Juan River Basin, Aguas Claras River, tributary of the Tatamá River. Scale 1 cm.

**Figure 2.**
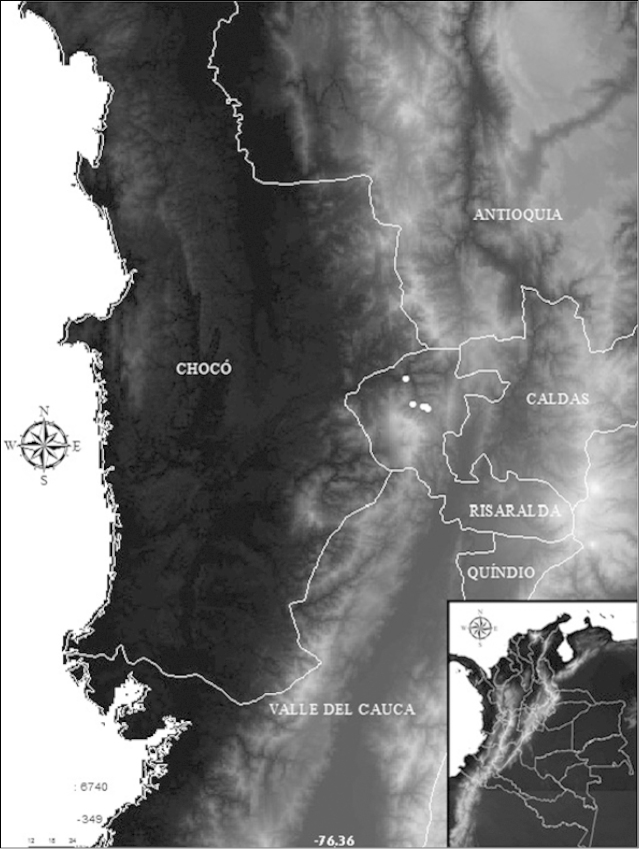
Distribution and location of *Hemibrycon sanjuanensis* sp. n. in the Alto San Juan River, Risaralda state, Colombia.

**Figure 3.**
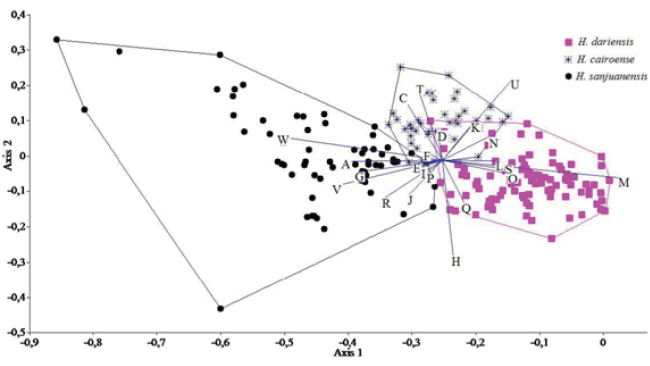
Representation of the Canonical Variables (CV canonical variable 1 is the X axis and canonical variable 2 is the Y axis) for *Hemibrycon sanjuanensis* sp. n., *Hemibrycon cairoense* and *Hemibrycon dariensis*.

**Figure 4.**
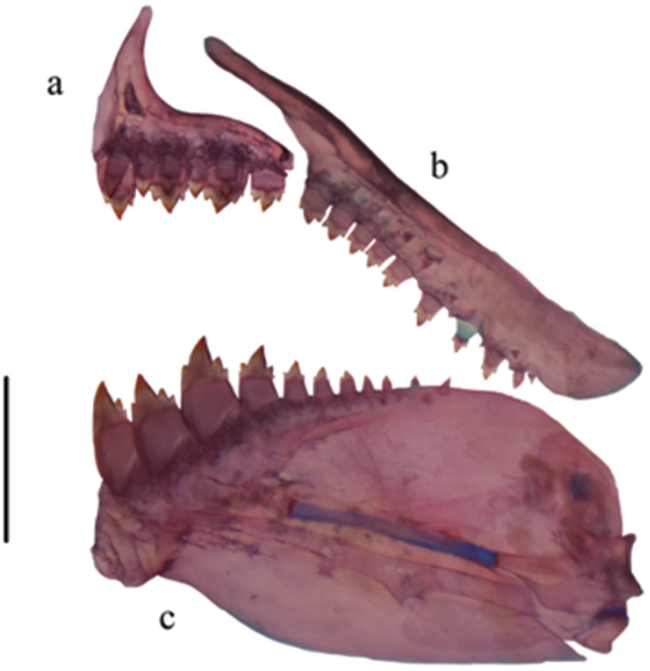
Upper and lower jaws of *Hemibrycon sanjuanensis* sp. n. IUQ 3695, 55.5 mm SL, A Premaxilla B Maxilla C Lower jaw. Scale bar = 1 mm.

Table 1 –2 , Figures 1 , 2 , 3 , 4

#### Holotype.

IUQ 3693, 53.5 mm SL, Colombia, Risaralda, Pueblo Rico, El Recreo, upper San Juan River, Aguas Claras River, Tatamá River tributary on Apia-Pueblo Rico road, 5°13'04.9"N, 76°01'50.1"W; 1519 m.a.s.l. (meters above sea level); **Paratypes:** IUQ 3039, 8, 47.0-58.4 mm SL, collected with holotype; IUQ 3040, 8, 39.3-81.2 mm SL, Risaralda, Pueblo Rico, La Selva, upper San Juan River Basin, La Selva Creek, tributary of Taiba River, 5°14'29.1"N, 76°04'42.1"W; 1359 m.a.s.l; IUQ 3041, 2, 70.6–84.9 mm SL, Risaralda, Pueblo Rico, upper San Juan River Basin, creek 1 km from Pueblo Rico on road to Santa Cecilia, 5°14'06.1"N, 76°02'20.1"W; 1357 m.a.s.l; IUQ 3042, 17, 21.3–70.9 mm SL, Risaralda, Pueblo Rico, upper San Juan River Basin, Agua Bonita Creek tributary of Tatamá River on road from Pueblo Rico to Santa Cecilia, 5°13'46"N, 76°02'05.1"W; 1530 m.a.s.l.; IUQ 3694, 2 C and S, 66.0–69.3 mm SL, Risaralda, Pueblo Rico, La Selva, upper San Juan River Basin, La Selva Creek, tributary of Rio Taiba, 5°14'29.1"N, 76°04'42.1"W; 1359 m.a.s.l; IUQ 3695, 2 C and S, 55.0–66.7 mm SL, Risaralda, Pueblo Rico, upper San Juan River Basin, Agua Bonita Creek, tributary of Tatamá River on Pueblo Rico- Santa Cecilia road, 5°13'46"N, 76°02'05.1"W; 1530 m.a.s.l.; IUQ 3697,19, 57.5–82.2 mm SL, Risaralda, Pueblo Rico, El Indio on Pueblo Rico-Villa Claret road, 200 m upstream from bridge, upper San Juan River Basin, Tatamá River, 5°1'50.52"N, 76°0'9.36"W; 1407 m.a.s.l.

#### Diagnosis.

*Hemibrycon sanjuanensis* sp. n. differs from *Hemibrycon boquiae*, *Hemibrycon brevispini*, *Hemibrycon cairoense*, *Hemibrycon colombianus*, *Hemibrycon mikrostiktos*, *Hemibrycon metae*, *Hemibrycon palomae*, *Hemibrycon rafaelense* and *Hemibrycon tridens* by the presence of a circular or oblong humeral spot located two scales posterior to the opercle (vs. 3–4 scales in *Hemibrycon palomae*, *Hemibrycon rafaelense*, *Hemibrycon brevispini* and *Hemibrycon cairoense*, and 0–1 scales in *Hemibrycon metae* and *Hemibrycon boquiae*). It further differs from *Hemibrycon colombianus* in having a round or oblong humeral spot (vs. rectangular). It differs from *Hemibrycon beni*, *Hemibrycon dariensis*, *Hemibrycon divisorensis*, *Hemibrycon helleri*, *Hemibrycon huambonicus*, *Hemibrycon inambari*, *Hemibrycon jabonero*, *Hemibrycon jelskii*, *Hemibrycon mikrostiktos*, *Hemibrycon polyodon*, *Hemibrycon quindos*, *Hemibrycon raqueliae*, *Hemibrycon santamartae*, *Hemibrycon surinamensis*, *Hemibrycon taeniurus*, *Hemibrycon tridens* and *Hemibrycon yacopiae* in having melanophores present on the posterior margins of the scales all along the sides of body (vs. melanophores absent from margins of scales along entire length of sides of body). The new species further differs from all the species mentioned above in having a wide, concave pelvic bone (vs. narrow and straight); the middle part of the dorsal margin of the orbito-sphenoid bone flattened and not in contact with frontal (vs. dorsal margin straight and in contact with frontal); ventral tip of supracleithrum bifurcate (vs. not bifurcate); six teeth in the outer premaxillary row arranged in a straight line (vs. five or fewer teeth in outer premaxillary row and not arranged in straight line, except *Hemibrycon cairoense* with two to six teeth in the outer premaxillary row).

#### Description.

Body slender and elongate (mean maximum body depth about 26.1% SL). Area above orbits flat between anterior margin of orbits and supraoccipital spine. Dorsal profile of head and body oblique from supraoccipital to dorsal-fin origin and from last dorsal-fin ray to base of caudal fin. Ventral profile of body convex from snout to base of pelvic fin; straight from pelvic-fin origin to anal fin. Caudal peduncle laterally compressed. Head and snout short (21.2% SL and 25.0% HL respectively), jaws equal, mouth terminal, lips soft and flexible, and outer row of premaxillary teeth; ventral border of upper jaw flat; posterior edge of maxilla reaching anterior edge of orbit. Premaxilla with two rows of teeth (Fig. 4). Six teeth of outer row tricuspid with central cusp largest, teeth arranged in straight line. Inner premaxillary row with four pentacuspid teeth that diminish gradually in size. Maxilla long, posterior margin straight, with 5–11 uni- or tricuspid teeth, central cusp slightly longer than outer cusps in tricuspid teeth (Fig. 4). Dentary with three or four large tricuspid teeth with central cusp largest, followed by two to four smaller, uni- to tricuspid teeth (Fig. 4). Six infraorbitals present, the first thin and narrow, extending between the dorsal edge of maxilla and lateral ethmoid, with sensorial canal. Second infraorbital short and wide, not covering dorsal part of angulo-articular. Anterior part of second infraorbital overlaying anterior part of first infraorbital; its posterior margin extending below third infraorbital. Third infraorbital widest and longest, its ventral border in contact with sensorial canal of preopercle. Fourth, fifth and sixth infraorbitals short and wide, covering posterior margin of hyomandibular. Supraorbital absent. Eight to nine supraneurals present between head and anterior part of dorsal fin, without cartilage on upper and lower edges, and with medial sensorial canal. Scales cycloid, moderately large. Lateral line complete with 40–47 pored scales (44*, mean=42.5, n=40). Scale rows between dorsal-fin origin and lateral line 5–6 (5*, mean=5.9); scale rows between lateral line and pelvic-fin origin 4–6 (5*, mean=5, n=40); scale rows between lateral line and anal-fin origin 4–6 (5*, mean=4.8, n=40); predorsal scales 11–14, arranged in regular series 11–14 (12*, mean=12.4, n=40). Dorsal-fin rays iii, 7–8 (iii, 7*, n=40); first unbranched ray approximately one-half length of second ray, its tip not reaching first bifurcation of first branched ray. Anal-fin rays iii-iv, 24–30 (iii, 27*, n=40). Anal-fin origin posterior to vertical through base of last dorsal-fin ray. Pectoral-fin rays ii, 9–12 (11*, n=40). Pelvic-fin rays ii, 6 (6*, n=40). Pelvic-fin origin anterior to vertical through dorsal-fin origin. Caudal fin not covered with scales except at its base, forked with short pointed lobes. Total number of vertebra 39–41.

**Table 1.** Counts and measurements of *Hemibrycon sanjuanensis* sp. n. Standard and total lengths in mm, averages in parentheses.

	Holotype	Paratypes
Standard length	53.3	21.3–84.9 (60.0)
Total length	63	6.3–100.3 (71.1)
Percentages of SL		
Body depth	26.4	19.2–30.2 (26.1)
Snout-dorsal fin distance	50.7	23.3–53.5 (49.7)
Snout-pectoral fin distance	24	21.7–27.3 (23.0)
Snout-pelvic fin distance	41.5	41.4–47.5 (42.9)
Snout-anal fin distance	56.8	54.4–61.1 (56.6)
Dorsal fin-hypural distance	53.7	26.7–57.4 (51.5)
Dorsal-fin length	20.1	18.3–24.4 (20.4)
Pectoral-fin length	19.9	15.3–20.5 (18.5)
Pelvic-fin length	12.6	8.6–13.3 (11.7)
Caudal peduncle depth	11.8	5.3–12.3 (10.7)
Caudal peduncle length	12	5.6–13.6 (10.7)
Head length	21.7	18.9–26.2 (21.2)
Dorsal-anal fin distance	27.6	22.8–38.0 (27.9)
Dorsal-pectoral fin distance	37.7	34.2–43.5 (38.3)
Anal-fin length	15.8	11.0–17.5 (13.8)
Percentages of HL		
Snout length	25	16.0–29.0 (25.0)
Orbital diameter	37.3	29.5–44.2 (36.2)
Postorbital distance	34.2	29.1–44.9 (38.8)
Maxilla length	38.2	25.7–41.6 (33.7)
Interorbital distance	38.5	32.0–44.7 (36.8)
Mandible superior distance	30.8	25.7–37.4 (32.8)
Lateral-line scales	44	40–47
Scale row between dorsal-fin origin and lateral line	6	5–6
Scale rows between anal-fin origin and lateral line	5	4–6
Scale rows between pelvic-fin and lateral line	5	4–6
Predorsal median scales	12	11–14
Dorsal-fin rays	iii, 7	iii, 7–8
Anal-fin rays	iii, 27	iii-iv, 24–30
Pelvic-fin rays	ii, 6	ii, 6
Pectoral-fin rays	ii, 11	ii, 9–12
Maxillary teeth	5	7–10

#### Secondary sexual dimorphism.

Males have between 3–11 very short hooks present on all branched pelvic-fin rays, located on both branches of rays, but predominantly on internal and lateral branches; hooks present on one simple pelvic-fin ray. Males have row of very short hooks on first seven to fifteen branched anal-fin rays, each ray has from 3–5 hooks, which extend along the extreme distal branch of rays; no hooks on simple anal-fin rays.

#### Live colors.

Dorsum of body and head silvery green; sides and ventrum silvery white from opercle to caudal peduncle. Caudal peduncle with dark midlateral stripe that extends on to middle caudal-fin rays and has a reddish spot on the ventral portion of the caudal- fin base. Humeral spot dark and rounded or oblong with some dispersed melanophores extending dorsally and ventrally. Pectoral and pelvic fins hyaline, but dorsal, anal and caudal fins with hues of reddish yellow and with dispersed melanophores on interradial membranes.

#### Pigmentation in alcohol.

Body dark brownish-yellow, melanophores more densely concentrated on dorsum and upper sides than on ventrum, most intense on head; with melanophores present on the posterior margins of the scales all along the sides of body. Trunk with dark, midlateral stripe from posterior margin of humeral spot to caudal peduncle, extending on to caudal fin. Humeral spot circular or oblong, located two scales posterior to the opercle, not reaching the first series of scales below the lateral-line canal. Ventral part of body light yellow. Dorsal fin with melanophores concentrated mostly on interradial membranes and rays. Adipose fin hyaline. Dark melanophores present on middle caudal-fin rays, near caudal-fin base. Pectoral and pelvic fins hyaline; anal and caudal-fin lobes dusky and with melanophores concentrated on interradial membranes to form band.

#### Distribution and ecological notes.

This species is so far known only from the upper San Juan River Basin, Tatamá River drainage, Pacific versant, Colombia (Fig. 2). *Hemibrycon sanjuanensis* sp. n. was captured in streams characterized by relatively rapid water running over rocky and sandy substrates with high transparency. The pH was near neutral, dissolved oxygen values were high, and conductivity and total solids were low (Table 2), typical of oligotrophic environments.

**Table 2.** Physicochemical variables in habitat of *Hemibrycon sanjuanensis* sp. n. San Juan River Basin, Colombia. Localities: 1. Aguas Claras River 2. La Selva Creek, 3. Agua bonita Creek. 4. Chupaderos Creek, 5. La Soledad Creek, 6. Itauri Creek, 7. Tatama River, m.a.s.l. = meters above sea level; Rd. Rocks and detritus; Rs. Rocks and Sand; width: of river at collecting site.

	Locality						
	1	2	3	4	5	6	7
m.a.s.l.	1519	1359	1530	1375	1219	446	1407
Water temperature (°C)	16.9	18.3	20.0	17.8	19.2	22.4	21.3
Air temperature (°C)	19.9	23.7	20.0	20.8	22.2	23.7	22.1
Dissolved oxygen (mg/l)	7.8	7.5	6.5	7.5	6.9	6.4	7.2
pH	7.51	7.47	6.3	7.6	7.57	7.67	7.57
Conductivity (us/cm)	0.49	0.446	0.001	0.698	-	1.443	0.72
Total solids (STD)	0	0	1	1	1	1	-
Width (m)	10	5–6	1–2	1–2	1–1.5	5–6	10.0–15.0
Depth (m)	1–2	0.5	0.5	0.3–0.5	0.5	0.5–1	0.5–3
Color	clear	clear	clear	clear	clear	clear	clear
Substrate	Rs	Rd, Rs	Rd, Rs	Rs	Rs	Rs	Rs

The new species is syntopic with *Bryconamericus emperador*, *Astroblepus* sp., *Pimelodella* sp. and *Characidium* sp. The analysis of stomach contents of four specimens revealed the presence of adults and larvae of two different species of Coleoptera: Hydrophilidae, *Promoresia* sp. and *Elmid* sp., Diptera: Simulidae and Sarcophagidae, adult of Odonata Zygoptera, Dytiscidae, Trichoptera, Nematoda, Isopoda and fragments of unidentified arthropods. The presence of autochthonous and some allochthonous items suggest that this species is insectivorous with considerable plasticity in its diet.

#### Etymology.

*Hemibrycon sanjuanensis* is named for the San Juan River Basin, where the type series was collected (Fig. 2).

#### Remarks.

Canonical Variables Analysis (CVA) of *Hemibrycon sanjuanensis* and species found in the San Juan and nearby rivers (including *Hemibrycon dariensis* and *Hemibrycon cairoense*), revealed significant differences among them, based on several characters, the most important of which are the length of the pelvic fin (variable M) and upper jaw length (related to variable W) (Fig. 3); the first canonical variable explained 84.3% of total variation, the second explained 15.6%. Linear regression analysis of the upper jaw length showed positive increment of these variables in *Hemibrycon sanjuanensis* (vs. negative increment in the other species studied) and so separated it from the other species included in the analysis (r=-0.15, P=0.02).

Moreover, this new species is distinguished from *Hemibrycon cairoense* (Román-Valencia and Arcila-Mesa 2009) by the number of unbranched dorsal-fin rays (iii vs. ii), by the number of scales rows between the dorsal-fin origin and the lateral line (5–6 vs. 6–7), between the anal-fin origin and the lateral line (5–6 vs. 6–7), and between the pelvic-fin insertion and the lateral line (4–6 vs. 6–7), by the presence of hooks on the males (only on the pelvic fin rays vs. on the anal, pelvic, pectoral and posterior portions of dorsal fin ray).

#### Discussion.

*Hemibrycon sanjuanensis* has all of the synapomorphies observed in other *Hemibrycon* (Arcila-Mesa 2008, Mirande 2010). Although species of *Hemibrycon* have a similar color pattern throughout their geographic distribution, subtle differences in the concentration and distribution of black pigment in the humeral and caudal regions have been used to diagnose new species (Bertaco and Malabarba 2010; Román-Valencia et al. 2009a, b, c, 2013; Román-Valencia and Arcila-Mesa 2010).

*Hemibrycon sanjuanensis* has a reddish spot on the ventral portion of the caudal- fin base in life. This characteristic has also been observed in several other species of *Hemibrycon* (Bertaco et al. 2007; Román-Valencia et al. 2010) and has been suggested as a synapomorphy for the genus by Arcila-Mesa (2008) and Román-Valencia et al. (2013).

Two species, *Hemibrycon dariensis* and *Hemibrycon microformaa*, have been reported previously from the San Juan, Atrato and Leon River drainages (Román-Valencia and Ruiz-C 2007; Román-Valencia and Arcila-M. 2008). The new species is similar and probably related phylogenetically to *Hemibrycon dariensis* but is distinguished from it by the following: the presence of a dark lateral stripe that continues on to the middle caudal-fin rays (vs. wide lateral band extending only to bases of middle caudal-fin rays); pectoral fins not reaching pelvic-fin origins (vs. pectoral fins reaching pelvic-fin origins); 39–41 total vertebrae (vs. 37–38); mean length of pelvic fins 11.7% SL, (vs. 15.2% ). From *Hemibrycon microformaa* (Román-Valencia and Ruiz-C 2007), the new species differs in attaining a larger adult size (greater than 31 mm SL vs. SL < 31 mm); in not having flat dorsal margins of the orbits and ventral profile of the snout (vs. flat); terminal mouth with upper jaw not surpassing lower (vs. mouth subterminal); greater total number of vertebrae 39–41 (vs. 33–34); and a greater number of branched anal-fin rays 24–30 (vs. 14–16).

#### Comparative material examined.

*Hemibrycon beni*: UMSS 09585, 18, Bolivia, Amazonas/Madera/Beni/Bopo, Pekheñkhara River Imanblaya, 3 Jan. 1990; UMSS 0890, 50, Bolivia, Amazonas/Beni/Madera/Kaka, tributary of Taipiplaya-Taipiplaya Rivers, 3 Oct. 2008. *Hemibrycon boquiae*: All from Colombia, Quindío state, Salento municipality, Upper Cauca River, Quindío River: IUQ 301a, 3, (C and S), Boquia Creek, 4°38'35"N, 75°75'11"W, 1819 m.a.s.l; IUQ 754,104, Boquia Creek (4°38'35"N, 75°75'11"W, 1819 m.a.s.l; IUQ 871, 15, Boquia Creek, 4°38'35"N, 75°75'11"W, 1819 m.a.s.l. *Hemibrycon brevispini* paratypes: IUQ 883,6, Colombia, Quindío state, Calarcá municipality, Upper Cauca River, Quindío River, Venada Creek, tributary of Santo Domingo River, Quebrada Negra road, 4°26'52"N, 75°41'02"W, 1278 to 1304 m.a.s.l; IUQ 1453,5 (C and S), Colombia, Quindío state, Calarcá municipality, Upper Cauca River, Quindío River, Quebrada Negra and Venada Creek, drainage of Santo Domingo River, 200 m along Quebrada Negra road after the bridge of the Santo Domingo River. *Hemibrycon cairoense* Paratypes: IUQ 537, 2 (C and S), Colombia, Risaralda state, El Cairo, Upper Cauca River, Los Ramirez Creek, tributary of La Italia Creek, road from Quinchia to El Cairo, 200 m beside the bridge; IUQ 537, 2 (C and S), Valle del Cauca state, El Cairo municipality, Upper Cauca River, Los Ramirez Creek, tributary of La Italia Creek, El Cairo, road from Quinchia to El Cairo, 200 m from bridge. *Hemibrycon colombianus*: IAvH 3130, 28, Colombia, Santander state, Magdalena River, Moniquira River, Suarez River, Sept 11 and 13, 1995; IAvH 2942, 6, Colombia, Boyacá state, Suarez River and El Cobre Creek; IAvH 3132, 24, Colombia, Boyacá state, Moniquira and Suarez Rivers, El Cobre Creek; ICNMNH 755, 7, Colombia, Santander state, Capitanejo and Nevado Rivers. *Hemibrycon coxeyi* ANSP 70155 (Holotype), Ecuador, Hacienda Las Mascotas, mouth of the Pastaza River, Marañón River drainage. *Hemibrycon dariensis*: USNM 260697,1, Colombia, Choco state, Atrato River, Bernal Creek, tributary of Negua River, 17 Mar 1967; USNM 293218, 2, Panamá, of Kuna Yala, Madinga River between Pingandi River and Mandinga (Atlántico), 09°28'N, 70°06'W, 3 Mar 1985; USNM 293234, 1, Panamá, Darién, Pirre River about 1/2 km above El Real (Tuira River), Pacific,19 Feb 1985; USNM 293245,28, Panamá, Darien, Tuira River, Darién Province, Pucuro River about 3–4 km above the confluence of the Tuira River, Pacific, 17 Feb 1985; IUQ 523, 26, Colombia, Antioquia state, Uraba, Zungo River highway, León River system,17 Dec 1990; IUQ 524,2, Colombia, Antioquia, Uraba, Creek km 25 Mutatá-Chigorodo road, Dec 1990; IUQ 525, 26, Colombia, Antioquia, Uraba, Caribbean sea, León River drainage, Villarteaga River, Dec 1990. *Hemibrycon fasciatus* (paratypes): All from the state (Departamento) of Antioquia, middle Magdalena River Basin: IUQ 3065, 4; 32.4–71.5mm SL, Santa Gertrudis Creek, tributary of Nare River, Concepción municipality 6°19'21.0"N, 75°09'38.6"W, 1820 m.a.s.l., 31 Jan 2010; IUQ 3156, 1, 52.7mm SL, Santa Gertrudis Creek, tributary of Nare River, Concepción and Alejandría municipality, after the Peñol-Guatapé dam. *Hemibrycon guppyi*: BMNH 1906623, lectotype, 1. USNM 290406, 1, C and S. USNM 290406, 7. *Hemibrycon helleri*: (see Arcila-M., 2008). *Hemibrycon jelskii*: MUSM 36126, 7, Peru, Cusco state, Amazon River, La Convención, Echarate, Urubamba, Perotoni River, 28 May 2009; MUSM 35492, 13, Peru, Ucayali state, Amazonas, Atalaya, Sepaliva, Lazaro Creek tributary of Mishahua River, 28 May 2009. *Hemibrycon jabonero*: All from Venezuela: EBRG 7542, 10, Falcón state, Sierra San Luis, Mitare River on old road between Cabure and San Luis, 28 Jun 1996; IUQ 553, 3, Carabobo state, Valencia Lake basin, El Ercigue river, north of San Joaquin, 4 May 1991; MBUCV 22854, 18, Carabobo state, Valencia Lake, 18 Jun 1968; MBUCV 12530, 4, Miranda state, Grande River 500 m after Santa Cruz River, Guatopo National Park, 1 Mar 1981; MBUCV 9736, 1, 66.4 mm SL, Monagas state, San Juan River, Los Morros Caripito, 28 Apr 1977; MBUCV 27652, 4, Carabobo state, Grande River, Guatopo National Park, 20 Dec 1980; MBUCV 12514, 5, Miranda state, Grande River, 500 m after Santa Cruz River, Guatopo Nacional Park, 3 Feb1981; MBUCV 22809, 5, Carabobo state, Miquita River, 2 km south of Goaigoaza, 2 Dec 1969; MCNG 42720, 17, Merida state, Chama River, Lake Maracaibo Basin, 14 Nov 2005; MCNG 33313, 1, Zulia state, Río Chama, 3 km from bridge at road, 14 Nov 2005; MCNG 27567, 1, Lara state, Caño Los Giles, 5 km. S.W. La Pastora, upper Tocuyo River, Caribbean Sea, 1 Jan 1992; MCNG 24838, 12, Zulia state Merida-Tachira, Maracaibo basin, Escalante River, 3 Mar1991; MCNG 48498, 38; Portuguesa state, creek north of Ospino, road to Estación La Reinosa, 3 Nov 1995; MCNG 188, 1, Zulia state, Negro River, Lake Maracaibo Basin, 14 Nov. 2005; MCNG 7475, 2, Merida state, road to Tovar, Uribante, 14 Nov 2005; MCNG 24821, 3, Merida state, creek ca. 3 km. East from Capazón, 1 Jul 1991; EBRG 4324, 23, Aragua state, Limón River, pool 350 m NW of Profauna building, El Limón, 21 Mar 1990; EBRG 9950, 2, Carabobo state, Salado River, Hacienda Urama II, 2 Jul 2004; EBRG 9349, 4, Cojedes state, road to Hato Piñero, Las Damitas Creek, under bridge, 21 Mar 1990; EBRG 4324, 33, Aragua state, Limón River, pool 350 m NW of Profauna, El Limón, 21 Mar 1990; EBRG 9953, 32, Carabobo state, Morón River, old highway to Morón Reservoir, 30 Jun 2004; EBRG 9951, 20, Carabobo state, Alpargaton River, above quarry, 13 Apr 2005; MCNG 54541, 25, Falcon state, El Hueque Creek, bridge on Churuguara-Coro road, 14 Nov 2005; MCNG 54589, 3, Yaracuy state, creek on Hacienda Guaquira, 14 Nov 2005; MCNG 54610, 20, state Yaracuy, Sarare Creek 1 km from Hacienda Corozal, 14 Nov 2005; MCNG 54566, 46, Carabobo state, El Samán on Alpargaton River, above sand quarry, 14 Nov 2005; MCNG 54602, 16, Yaracuy state, Hacienda Guaquira, upper Guaquira Creek at cement crossing, 14 Nov 2005; MCNG 49640, 5, Crucito River, Crucito Sector, 1 km from asphalt de road to Palma Sola, 16 Jan 2004; MCNG 16972, 8, Cocotal River (or Cocollar) at Campo Elias before San Antonio, at mouth with Guarapiche River, San Juan River system, 14 Nov 2005; MCNG 6475, 2, Apure state, mouth of creek tributary to Grande River, Tachira River drainage, 14 Nov 2005. *Hemibrycon metae*: IAvH 3122, 10, Colombia: Casanare state, Aguazul, Orinoco, Chichaca Creek, tributary of Cachiza River, 1 Mar 1994; IAvH 3125, 33, Colombia: Casanare state, Aguazul, Orinoco, Unete, Cravo Sur and Tua River drainage, 4 May 1996; IAvH 3129, 50, Colombia: Casanare state, Aguazul, Orinoco, Cupiagu Creek, Unete River drainage, 4 Mar 1994; all from Venezuela: MCNG 26774, 2, Barinas state, Santa Barbara River, 3 km NE, Santa Barbara, Apure River basin, 1 Jan 1992; MCNG 26774, 26, Barinas state, Santa Barbara River, 3 km NE Santa Barbara, Apure River basin, 1 Jul 1992; MCNG 7916, 1, Barinas state, Apure, Pedraza District, Ticoporo Creek at bridge on road from Acequia River, 7 Dec 1982; MCNG 50011, 1, Ventuari River, Tencua Falls, 58 km. E of San Juan de Manapiare, 5°2.86'N, 65°36.95'W, 21 Apr 2004; MCNG 41903, 2, Barinas state, upper La Yuca River, 3 Nov 1998; MCNG 32396, 30, creek NE of San Antonio, Highway 5, Curito River, 3 Feb1993. *Hemibrycon surinamensis*: (see Arcila-Mesa 2008). *Hemibrycon tridens*: (see Arcila-Mesa 2008). *Hemibrycon micromorfaa* IUQ 512 (1 Paratype),(C and S), Colombia, Atrato River Basin, Chintado River, 100 m bridge on Yuto-Certegui; IUQ 1204,1, paratype, (C and S), Atrato River Basin, Chintado River, 100 m bridge on the Yuto-Certegui. *Hemibrycon mikrostiktos* (see Bertaco and Malabarba 2010). *Hemibrycon orcesi* ANSP 75904 (paratype), 2, Ecuador, Santiago-Zamora, headwaters of Macuma River, tributary of Morona River, 550–650 m.a.s.l. MEPN 001542, 2, paratype, Ecuador, Pastaza, Macuma River, Apr1953; MEPN 001538, 17, Ecuador, Morona-Santiago, Tayusa River, tributary of lower Upano River, at bridge on Méndez-Sucua road, 4 May 1991. MEPN 001539, 66, Ecuador, Morona-Santiago, Tayusa River, tributary of Upano River at bridge on Méndez-Súcula road, 4 May 1991; MEPN 001540, 10, Ecuador, Pastaza, downstream from Sarayaco, Aug 1956. MEPN 001541, 5, Ecuador, Pastaza, Pastaza River, AquaRAP, 22 Jul 1999. MEPN001543, 2, Ecuador, Pastaza, Bobonaza-Canelos River, Pastaza River Basin, Apr 1953. MEPN 13591–6, 39, Ecuador, Morona-Santiago, Tayusa River tributary Upano River 4, May 1991. *Hemibrycon pautensis*: MEPN 001550, 10, Ecuador, Carchi, El Voladero lagoons in El Angel Biological Reserve, 0°40'N, 77°52'W, 3680 m.a.s.l., 20 Jul 2001. *Hemibrycon palomae* (paratypes), all from Colombia, Cauca-Magdalena River Basin, Quindio, Quimbaya, El Ocaso natural reserve, Roble River drainage, La Paloma creek: IUQ 2300,6, 46.4–65.4 mm SL, 15 May 2008. *Hemibrycon polyodon* IUQ 1142, 2, (C and S), Ecuador, Antonio-Guadalupe Creek, 12 Mar 1979. *Hemibrycon quindos* paratypes: IUQ 487, 28, Colombia, Quindío state, Salento municipality, Llano Grande, Upper Cauca, Quindío River, Tinaja Creek 300 m from Llano Grande - Boquia road 4°36'57"N, 75°36'36"W, 1712m.a.s.l., 29 Jan. 2002; IUQ 488, 2 (C and S), Colombia, Quindío state, Salento municipality, Upper Cauca, Quindío River, Tinaja Creek, 300 m from Llano Grande - Boquia road, 4°36'57"N, 75°36'36"W, 1712 m.a.s.l., 29 Jan. 2002; IUQ 489, 15, Colombia, Quindío state, Salento municipality, Upper Cauca, Quindío River, Tinaja creek, 300 m from Llano Grande - Boquia road, 4°36'57"N, 75°36'36"W, 1712 m.a.s.l., 11 Jul. 1996; MTD F 27621–27622, 2, Colombia, Quindío, Salento, Llano Grande, Upper Cauca, Quindío River Basin, Tinaja creek, 300m from Llano Grande -Boquia road, 4°36'57"N, 75°36'36"W, 1712 m.a.s.l., 29 Jan. 2002. *Hemibrycon rafaelense*: (Paratypes) IUQ 509, 27, Colombia, Risaralda state, Apia municipality, Upper Cauca River, San Rafael Creek in mouth of Apia River, at 100 of Santuario-Apia Road,5°04'54"N, 75°56'36"W,1253 m.a.s.l., 8 Jul 2003; MCNG 54101, 5, Colombia, Risaralda state, Apia municipality, Upper Cauca River, San Rafael Creek at mouth of Apia River, 100 m from Santuario-Apia Road, 5°04'54"N, 75°56'36"W, 1253 m.a.s.l., 8 Jul 2003; MTD F 27623-27624,2, Colombia, Risaralda state, Apia municipality, Upper Cauca River, San Rafael Creek at mouth of Apia River, 100 m from Santuario-Apia Road,5°04'54"N,75°56'36"W, 1253 m.a.s.l., 8 Jul 2003. *Hemibrycon raqueliae* Paratypes: IUQ 496, 2, Colombia, Caldas state, Samaná municipality, Middle Magdalena River, La Miel River, Tasajo creek, 5°23'55"N, 74°59'05"W, 1482m.a.s.l., 3 Jan 2003; IUQ 497, 99, Colombia, Caldas state, Samaná municipality, La Vención, Middle Magdalena, La Miel River Basin, Santa Rita Creek, 4 Jan. 2003; MCNG 54102, 5, Colombia, Caldas state, Samaná municipality, La Vención, Middle Magdalena River, La Miel River, Santa Rita creek, 4 Jan. 2003; IUQ 498, 2 (C and S), Colombia, Caldas state, Samaná municipality, La Vención, Middle Magdalena River, La Miel River, Santa Rita creek, 4 Jan 2003; ICNMHN 3281, 5, Colombia, Caldas state, at Norcasia-Samaná road, Middle Magdalena River, La Miel River, El Aquila creek, 8 Dec. 1992; MHNUC 019, 1, Colombia, Caldas, state Manzanares municipality, Middle Magdalena River, Manzanares River, 5°03'06"N, 75°35'05"W, 7 Dec. 1997; MTD F 27625- 27626, 2, Colombia, Caldas state, Samaná municipality, La Vención, Middle Magdalena River, La Miel River, Santa Rita Creek, 4 Jan. 2003. *Hemibrycon taeniurus* All from Venezuela: EBRG 4541, 32 Sucre state, Paria Peninsula National Park, Los Mangos sector, Yoco River; EBRG4544, 47, Sucre state, Paria Peninsula National Park, Los Mangos sector, La Toma River. EBRG 4546, 39, Sucre state, Paria Peninsula National Park, Los Mangos sector, Bautista River; EBRG 4547, 17, Sucre state, Paria Peninsula National Park, Los Mangos sector, Las Cabreras Creek; EBRG 4550, 3, Sucre state, Paria Peninsula National Park, Los Mangos sector, Solis Creek; EBRG 4553, 2, Sucre state, PN Península de Paria, Las Melenas Sector, Río El Chispero (upper sector). EBRG 4556, 12, Venezuela, Sucre state, Paria Peninsula National Park, Los Mangos sector, Río Yoco. EBRG 4558, 7, Sucre state, Paria Peninsula National Park, Los Mangos sector, Río El Hoyo; EBRG 4560, 5; Sucre state, Paria Peninsula National Park, Los Mangos sector, Creek Las Cabreras; EBRG 4566, 12, Sucre state, Paria Peninsula National Park, Los Mangos sector, Río Guarama; EBRG 4569, 3, Sucre state, Paria Peninsula National Park, Los Mangos sector, Creek Solis. EBRG 4570, 2, Sucre state, Paria Peninsula National Park, Los Mangos sector, Creek Las Cabreras; EBRG 4571, 7, Sucre state, Paria Peninsula National Park, Los Mangos sector, Bautista River; EBRG 4581, 9, Sucre state, Paria Peninsula National Park, Los Mangos sector, Río Salado; EBRG 4584, 5, Sucre state, PN Península de Paria Las Melenas Sector, El Chispero River. EBRG 4605, 1, Sucre state, PN Península de Paria Las Melenas Sector San Antonio River about 200 m upstream from San Antonio; EBRG 4627, 2, Sucre state, PN Península de Paria Las Melenas Sector, Maraval River about 500 m upstream from Maraval. EBRG 8488, 45; Monagas state, El Guamo Dam, in cove east of MARN campsite. EBRG 8599, 1, Anzoategui state, Amana River, river crossing on Mundo Nuevo Road. EBRG 8708, 5, Monagas state, El Guamo dam below the spillway, 10°05'25"N, 63°39'20"W; EBRG 8712, 1, Monagas state, El Guamo dam beside spillway,10°05'46"N, 63°39'02"W; EBRG 8714, 12, Monagas state, Negro River tributary from Guarapiche River, 10°09'23"N, 63°41'27"W; EBRG 8915, 1, Venezuela, Sucre state, Colorado River at bridge near transfer tunnel of treatment plant, 10°10'39"N, 64°18'02"W; EBRG 8916,2, Sucre state, Turimiquire Dam at tunnel outlet,10°10'38"N, 64°17'59"W; EBRG 8925, 7, Sucre state, Colorado River, tributary of Neveri River, 10°10'42"N, 64°18'06"W; EBRG 8970, 18, Sucre state, Turimiquire Mountain Range, Santa Cruz River at Santa Cruz, 10°20'43"N, 63°34'33"W, 33 m.a.s.l.; MHNLS 8046, 2, Monagas state, Punceres River, 15 km from Quiriquire, 10°07'07"N, 63°9'W; MHNLS 8070, 119, Monagas state, Aragua River (Los Becerros Creek)at bridge, Maturín-Quiriquire road, 10 km from Aragua de Maturín, 63°25'W, 10°00'24'N, 100 m.a.s.l., 13 Feb 1991; MHNLS 8091, 72, Monagas state, Aragua River (Los Becerros Creek at bridge ), Maturin-Quiriquire road, 10 km from Aragua de Maturín, 63°25'W, 10°00'24'N, 100 m.a.s.l., 13 Feb 1991; MHNLS 8157, 52, Venezuela, Sucre state, Río Parare,5 km from Río Grande, Quiriquire-Cariaco road, 63°17'W, 10°19'N, 15 Feb 1991; MHNLS 8888, 191, Monagas, Aragua River (Los Becerros Creek at bridge ), Maturin-Quiriquire road, 10 km Aragua from Maturín, 63°25'W, 10°19'N; 100 m.a.s.l., 3 Feb 1991; MHNLS 8891, 6, Monagas state, Aragua River (Los Becerros Creek at bridge ), Maturín-Quiriquire road, 10 km. from Aragua de Maturín, 63°25'W, 10°19'N, 100 m.a.s.l., 3 Feb1991; MBUCV 5036, 76, Sucre state, La Toma, Carúpano, 18 Jun1967. *Hemibrycon santamartae* (Paratypes) all from Colombia in Cesar state, Rancheria River drainage: IUQ 924,1, Atanquez municipality, Sierra Nevada de Santa Marta, Candela River, approx. 11°15'N, 74°05'W; IUQ 929, 3, Magdalena River, Sierra Nevada de Santa Marta, Honduras Creek on road to Mutaiahi, approx., 11°15'N, 74°10'W; IUQ 1443,1 CandS), Atanquez municipality, Sierra Nevada de Santa Marta, Candela River, approx. 11°00'N, 72°46'W; ICNMNH 10834,18, La Guajira, Distracción municipality, Chorreras, bridge at Cercado, road from Distracción to Caracolí, approx. 11°15'N, 74°05'W; ICNMNH 10839, 19, Colombia, La Guajira, Marocaso municipality, approx. 11°15'N, 74°05'W. ICNMNH 10881, 24, La Guajira, Marocaso municipality, Marocaso River, approx. 11°15'N, 74°05'W. *Hemibrycon virolinica* (Paratypes): ICNMHN 6736, 12, Colombia, Santander, Charalá, Virolín, Luisito-Virolín Rivers, La Cristala Creek, 6°06'24"N, 73°11'55"W, 1759 m.a.s.l., 29 Nov. 1978; IUQ 521, 9, Colombia, Santander, Charalá, Virolin River, Cañaverales River on the Virolin-Sogamoso road, 6°05'40"N, 73°11'58"W, 1744 m.a.s.l., 4 Feb. 2004; MCNG 54103,2, Colombia, Santander, Charalá, Virolín Creek, Cañaverales River on the Virolin-Sogamoso road, 6°05'40"N, 73°11'58"W, 1744 m.a.s.l., 4 Feb. 2004; IUQ 522, 4, Colombia, Santander, Charala, Virolín Creek on the Virolin-Sogamoso road, 6°06'02"N, 73°11'35"W, 1790 m.a.s.l., 4 Feb. 2004. *Hemibrycon tridens* (see Bertaco and Malabarba 2010). *Hemibrycon yacopiae* (Paratype): IUQ 515, 4, Colombia, Cundinamarca, Yacopi, Hatico-Moral, Hatico River, Aldana River system, Middle Magdalena River Basin, 5°31'22"N, 74°19'30"W, 761 m.a.s.l., 26 Aug. 2003; IUQ 516, (8); Colombia, Cundinamarca, Yacopi, La Mina Creek at Yacopi-La Mina road, Magdalena River Basin, 5°25'51"N, 74°19'59"W, 1094 m.a.s.l., 27 Aug. 2003; MCNG 54104, 2, Colombia, Cundinamarca, Yacopi, La Mina Creek at Yacopi-La Mina road, Magdalena River Basin, 5°25'51"N, 74°19'59"W, 1094 m.a.s.l., 27 Aug. 2003; MTD F 26627, 1, Colombia, Cundinamarca, Yacopi, La Mina Creek at Yacopi-La Mina road, Middle Magdalena River Basin, 5°25'51"N, 74°19'59"W, 1094 m.a.s.l., 27 Aug. 2003.

## Supplementary Material

XML Treatment for Hemibrycon sanjuanensis
